# Hematopoietic Multipotent Progenitors and Plasma Cells: Neighbors or Roommates in the Mouse Bone Marrow Ecosystem?

**DOI:** 10.3389/fimmu.2021.658535

**Published:** 2021-04-15

**Authors:** Amélie Bonaud, Julia P. Lemos, Marion Espéli, Karl Balabanian

**Affiliations:** ^1^ Université de Paris, Institut de Recherche Saint-Louis, EMiLy, INSERM U1160, Paris, France; ^2^ OPALE Carnot Institute, The Organization for Partnerships in Leukemia, Hôpital Saint-Louis, Paris, France

**Keywords:** bone marrow, hematopoietic stem and progenitor cell niches, multipotent progenitors, plasma cells, lymphoid lineage, CXCR4, WHIM syndrome

## Abstract

The bone marrow is a complex ecosystem in which hematopoietic and non-hematopoietic cells reside. In this review, we discuss the bone marrow niches in mice that facilitate the survival, maintenance, and differentiation of cells of hematopoietic origin based on the recent literature. Our review places a special focus on the hematopoietic multipotent progenitors and on plasma cells, corresponding to the last stage of the B-cell lineage, that play a key role in the humoral memory response. We highlight the similarities between the microenvironments necessary for the establishment and the maintenance of these two immune cell subsets, and how the chemokine CXCL12/CXCR4 signaling axis contributes to these processes. Finally, we bring elements to address the following question: are multipotent progenitors and plasma cells neighbors or roommates within the bone marrow?

## Introduction

The bone marrow (BM) is a complex organ in which hematopoiesis takes place during adulthood. Both hematopoietic and non-hematopoietic cell types cohabit in the BM and form distinct environments capable of promoting cell differentiation and survival in response to the organism needs. In this complex ecosystem, hematopoietic stem and progenitor cells (HSPCs) co-exist with cells at various intermediate differentiation stages, including fully differentiated cells (e.g., plasma cells [PCs]) (1). In the adult mouse, all functional hematopoietic stem cell (HSC) activity is found within the Lin-Sca1+c-Kit+ (LSK) compartment, which comprises about 0.00125% of total BM cells. This small cellular population is itself subdivided into various subsets based on the expression of cell surface markers such as CD34, CD135 (Flk2/Flt3), CD150, and CD48 (2). Adult hematopoiesis is a thoroughly regulated process that is initiated from quiescent pluripotent HSCs, which encompass long-term HSCs (LT-HSCs) and short-term HSCs (ST-HSCs) (3, 4). Multipotent progenitors (MPPs) are the immediate progeny of HSCs. For several years, MPPs have been considered as a homogeneous population with limited to no self-renewal capacity – in contrast to the more immature CD34-CD150+CD48- HSC compartment –, but with multi-lineage differentiation potential toward the earliest myeloid and lymphoid progenitors in the hematopoietic tree. However, recent studies have shown that the MPP compartment is more heterogeneous than expected and can be divided into four distinct subsets with different lineage fates. MPP1, defined as CD150+CD48-CD135-, shares characteristics with ST-HSCs including multiple-lineage reconstitution ability (5, 6). At steady state, they presumably give rise to functionally distinct lineage-biased MPPs that are more proliferative, devoid of self-renewal potential, and defined as megakaryocyte/erythroid (ME)-biased MPP2 (CD150+CD48+CD135-), granulocyte/macrophage (GM)-biased MPP3 (CD150-CD48+CD135-), and lymphoid-biased MPP4 (CD150-CD48+CD135+) (4–7). Although it is assumed that transition of HSCs from quiescent to more proliferative states associated with differentiation requires a unique set of bioenergetics demands, little is known about the metabolic requirements of MPPs. The MPP compartment is also dynamic and functionally plastic. In particular, the lineage fate of MPPs seems to be not fixed and can be redirected under specific conditions. Accordingly, the Passegué laboratory showed that lymphoid-primed MPP4 with their intrinsic GM poising contributed to myeloid output at steady state and underwent a transient change in their molecular identity that redirected them away from lymphoid differentiation to participate, together with overproduced MPP2 and MPP3, in the burst of myeloid production in blood regenerative conditions (4). Moreover, increased myeloid differentiation is also observed during chronic or infectious diseases, such as chronic myelogenous leukemia and acute viral infection (8, 9), or during homeostatic processes such as aging. Indeed, the Trowbridge group recently reported a progressive loss of lymphoid-primed MPP4 with aging concomitant with expansion of HSCs (10). Apart from CXCL12 and IL-6, two factors released by mesenchymal stromal/stem cells (MSCs) that have been reported to regulate MPP homeostasis and maintenance (11–14), our understanding of how cell-extrinsic niche-related and cell-intrinsic cues drive the lymphoid versus myeloid fate decision of MPPs is still incomplete.

Although not fully understood, it is now well established that BM environmental cues are integrated by hematopoietic cells throughout their differentiation and translate into distinct cell fates. As a typical example, the chemokine CXCL12 produced by non-hematopoietic stromal cells is essential to promote Common Lymphoid Progenitor (CLP) differentiation toward the B-cell lineage (15), through the progressive differentiation of pro-B cells into pre-B cells and then into immature B cells (16, 17). This process is essential for efficient rearrangement of the immunoglobulin loci and production of a functional B-cell receptor (BCR) (18–21). B cells then pursue their maturation in the periphery and, upon activation during an immune response and notably through the formation of germinal centers, some B cells differentiate into PCs, which corresponds to the final differentiation stage of the B-cell lineage responsible for antibody (Ab) production. Most PCs are short lived but some of them can relocate into the BM where they mature into long-term PCs. This occurs in specific niches that support their survival, maintenance and dormancy through cellular and soluble factors and ensure long-term (potentially life-long) protection against reinfection. Whether newly produced PCs and long-lived PCs reside in and/or compete for identical niches is still unknown (22, 23).

In the last two decades, the notion of niches has become an essential part of how we envision the organization and function of the BM ecosystem. Although the definition of “niches” may vary depending on the studies, a unifying view is that they correspond to complex, dynamic microstructures in which several soluble and membrane-anchored factors are produced, allowing the correct positioning of a specific cell type to favor interactions with other cellular actors and access to all the elements necessary for their maintenance or differentiation (24). To date, the definition of what a niche should be is based on the analyses of essential elements constituting the survival and differentiation cues for HSCs. These niches are thought to be composed of perivascular mesenchymal units associated with sinusoids and arterioles (25–28). However, a great heterogeneity may exist in these cell populations and should be integrated into the definition(s) of a niche. Strikingly, the nature of the niche(s) supporting the differentiation and maintenance of other cell types, including MPPs and PCs, has not been studied in detail. In this review, we will discuss these niches, with emphasis on the essential cellular network needed for MPP and PC maintenance within the mouse BM. Based on current literature, we will delineate the roles played by specific stromal cells and various actors in the MPP and PC niches and highlight common factors necessary for the maintenance of these cell populations including the CXCL12/CXCR4 signaling axis. We will also open a discussion on three essential and unresolved questions: (1) Do specific niches or interaction networks exist for each MPP and PC subset?; (2) Do MPP and PC subsets share niches or compete for them?; and (3) Do MPP and PC subsets regulate their own niche or affect each other?

## The Vascular Versus Osteoblastic Niches: How Far From Reality?

Bones can be anatomically divided in four main categories: long, short, flat and irregular bones. The long bones can be structurally divided in the epiphysis, which is filled with spongy bone and red marrow, the diaphysis, a tubular shaft, lined with a dense and compact cortical bone, and the intermediate metaphysis, which contains the epiphyseal plate that allows bone growth (29). Moreover, it can be schematically divided in three major regions: the cortical bone that forms the hard-outer layer of bones, a central cavity containing the marrow, and the endosteum, which corresponds to the interface between the cortical bone and the marrow. The endosteum is enriched in fully committed bone-forming osteoblasts and bone-resorbing osteoclasts and spreads along the inner bone surface (29). Arteriolar vessels are found near the endosteum, longitudinally aligned along the diaphysis and supply oxygen, nutrients, and growth factors to the marrow. Most vessels within the marrow are specialized venules called sinusoids that form a dense network. The sinusoids finally merge into a central sinus to form the venous circulation (24). In addition to this spatial distribution, different types of BM endothelial cells (ECs) have been phenotypically identified in long bones and in flat and irregular ones as well (30). CD31loEndomucin(Emcn)lo L-type sinusoidal ECs are enriched in the marrow cavity, which is poorly occupied by CD31+Emcn- arteriolar ECs. Arteries co-stain for Sca-1 and CD31 and the distal smaller arterioles are surrounded by Sca-1+ mesenchymal and hematopoietic cells (26). On the contrary, the bone compartment is enriched for arteriolar ECs, few L-type sinusoidal ECs and CD31hiEmcnhi H-type ECs, a small fraction of the ECs at the end of the CD31+Emcn- arteriolar network (30, 31). The last ones were demonstrated to be in close contact with osteoprogenitor cells, providing niche signals that promote bone development and maintenance, besides healing after fracture (30, 32). These well-organized vascular structures, together with the cortical bone, have been classically used to discriminate osteoblastic/endosteal areas versus (peri)-vascular areas within the BM. The recent identification of transcortical vessels crossing the bone cortex (33) suggests that former concepts based on the dichotomy of the osteoblastic and vascular niches are not absolute and led to the reconciling concept of endosteo-vascular niches (25, 26). Indeed, perivascular units likely integrate contributions from ECs and osteoprogenitor cells, including MSCs and perisinusoidal stromal (PSS) cells, as well as from fully differentiated osteoblasts, leading to a more complex definition of the HSC niches (26, 34, 35).

Recently, the use of transgenic mice allowing the tracking of specific cell lineages, associated with single-cell RNA-seq analyses, has permitted to better characterize the diverse components of the BM environment and the heterogeneity of the cell types composing the hematopoietic niches (36–40). Moreover, imaging techniques that allow simultaneous mapping of the HSPCs and interacting stromal cells have been critical for the discovery of BM niches (36–39, 41, 42). Taken together, these key results suggest that HSC niches constitute a critical spatio-temporal regulatory unit composed of multiple mesenchymal, hematopoietic, and neuronal cell populations associated with different vessel subtypes that cross-interact in a highly dynamic setting and where exchange of key signals leads to multi-directional regulation of the different partners. Of note, most of these findings were made using mouse models and extra-caution is required when extrapolating these data to human in which the BM composition might be different (*e.g.*, the human BM contains noticeably more adipocytes). Because the roles of several cell types and many soluble and surface-anchored factors in the BM have been broadly studied and reviewed recently (17, 43–48), we will focus on how the different BM niches can regulate and potentially determine HSC fate and differentiation into lineage-biased MPPs in mice. Moreover, we will discuss whether these early progenitors may share some niche elements with differentiated mature cells, namely PCs.

## HSC and MPP Niches: Distinct or Shared?

HSCs are distributed throughout the BM and, under physiological conditions, around 30% of them are in a quiescent stage. Histological and functional assays first indicated that these slow-cycling LT-HSCs preferentially localize in endosteal and sub-endosteal regions, in association with the osteoblasts and the bone surface (49–51). Subsequent studies suggested that mature osteoblasts have an indirect effect on HSC activity and maintenance (52–55), whereas osteoprogenitor cells control HSPC survival and commitment. In particular, depletion of the rare peri-arteriolar osteoprogenitors (also called pericytes) changes the spatial location of HSCs, which move away from the arteries and acquire a non-quiescent state, thereby increasing the proportion of proliferative cells (51, 56). Therefore, most of the fast-cycling proliferative ST-HSCs, committed progenitors, and differentiated cells are distributed in the central region and predominantly localize next to sinusoids (52, 57, 58). This suggests that HSCs require different perivascular niches based on their cell cycle status and that specific endothelial or perivascular reticular/mesenchymal cells orchestrate this process. The enrichment of HSCs in contact with sinusoidal endothelium may ensure a more efficient hematopoietic cell mobilization (52, 59, 60). Several niche factors have been reported to provide HSCs with instructive clues to regulate their location, retention, self-renewing, and fate (57, 61–65). In particular, Stem Cell Factor (SCF) and CXCL12 are required for HSC maintenance and retention in the BM (17, 63, 66, 67). PSS cells identified by surface expression of the Leptin receptor (LepR) promote HSC maintenance and the use of knock-in mouse models showed that these cells are the main source of SCF and CXCL12 (31, 57, 62, 63, 67–69). SCF is present in both membrane-bound and soluble forms, and specific deletion of this factor from PSS cells decreases the numbers of HSC in the BM (28, 56, 63, 69). Similarly, CXCL12 is a chemokine required for HSC retention and localization in the BM, and CXCL12 deletion from PSS cells reduces HSC numbers, while impacting their quiescence status and their distribution (35, 65, 67).

Despite these breakthroughs, it is still unclear whether lineage-biased HSCs and MPPs are broadly distributed through the BM or occupy specific niches. Consistent with this idea, it was shown that platelet and myeloid-biased von Willebrand factor-positive (vWF+) HSCs, which also express high levels of CD150, reside in close association with megakaryocytes in the BM. Megakaryocyte depletion leads to vWF+ HSC expansion, loss of their long-term self-renewal capacity, and lineage-bias after transplantation, suggesting that megakaryocyte-enriched niches promote HSC quiescence as well as commitment (70–72). On the other hand, vWF- lymphoid-biased HSCs are rather enriched in arteriolar niches, and depletion of peri-arteriolar Neuron-glial antigen 2-positive stromal cells significantly reduces this population with no effect on the myeloid-biased cell numbers (72). Despite our partial understanding of the exact location of lineage-biased HSPCs, it is clear that their positioning within the BM plays a critical role in directing which lineage-specific signals are received by hematopoietic precursors.

In line with this, some studies suggest that MPPs reside further into the endosteal surface, in more perfused areas (59, 73), whereas others indicate that lymphoid specification of MPPs occurs in parallel to their migration away from the endosteal region, which is regulated by G protein-coupled receptors (GPCRs) (74). This suggests that the myeloid versus lymphoid specification of MPPs may occur in different locations within the BM and under different, specific conditions. More recently, CXCR4 expression was shown to be required for proper localization and differentiation of lineage-specific precursors. Indeed, Cordeiro-Gomes et al. showed that conditional deletion of CXCR4 in MPPs reduced their differentiation into CLPs and decreased lymphopoiesis, with a dramatic reduction in B, T, and natural killer cell production and, to a lesser extent, in myeloid progenitors (69). The CXCL12/CXCR4 signaling was not intrinsically required for *in vitro* lymphoid development from CLPs, but for promoting CLP positioning close to IL-7+ cells and proper IL-7R activation *in vivo*. Therefore, MPPs share with HSCs the requirement for CXCL12 signaling and may reside in similar niches, but in closer association with IL-7+ cells. Indeed, depletion of CXCL12 or SCF expression from IL-7+ cells reduced both HSC and MPP numbers in the BM (17, 69). Further insights into the role of the CXCR4/CXCL12 signaling axis on HSPC lineage commitment come from the study of a rare immunodeficiency called the WHIM (warts, hypogammaglobulinemia, infections, and myelokathexis) syndrome (14, 75–77), that leads to severe chronic pan-leukopenia. Most of the patients present an autosomal-dominant mutation in CXCR4, associated with receptor desensitization resistance and consequently gain of function in response to CXCL12 (78–81). Using an original knock-in mouse model of the WHIM syndrome (82), Freitas et al. reported that the profound circulating lymphopenia was associated with a decrease in lymphoid-primed progenitor numbers in the BM with no difference in the number of myeloid progenitors (14). Thus, efficient CXCR4 desensitization regulates lymphoid differentiation of HSPCs in the BM, and absence of this regulatory mechanism likely contributes to the lymphopenia observed in the mutant mice and likewise in the patients. This study identifies MPPs as the key hematopoietic stage at which CXCR4 signaling termination impacts lymphoid, but not myeloid, lineage commitment. Altogether, these works suggest a pivotal role for GPCRs, including CXCR4 signaling, in regulating the fate of MPPs, for which the BM niches and their precise localization are still largely unknown.

**Figure 1:**
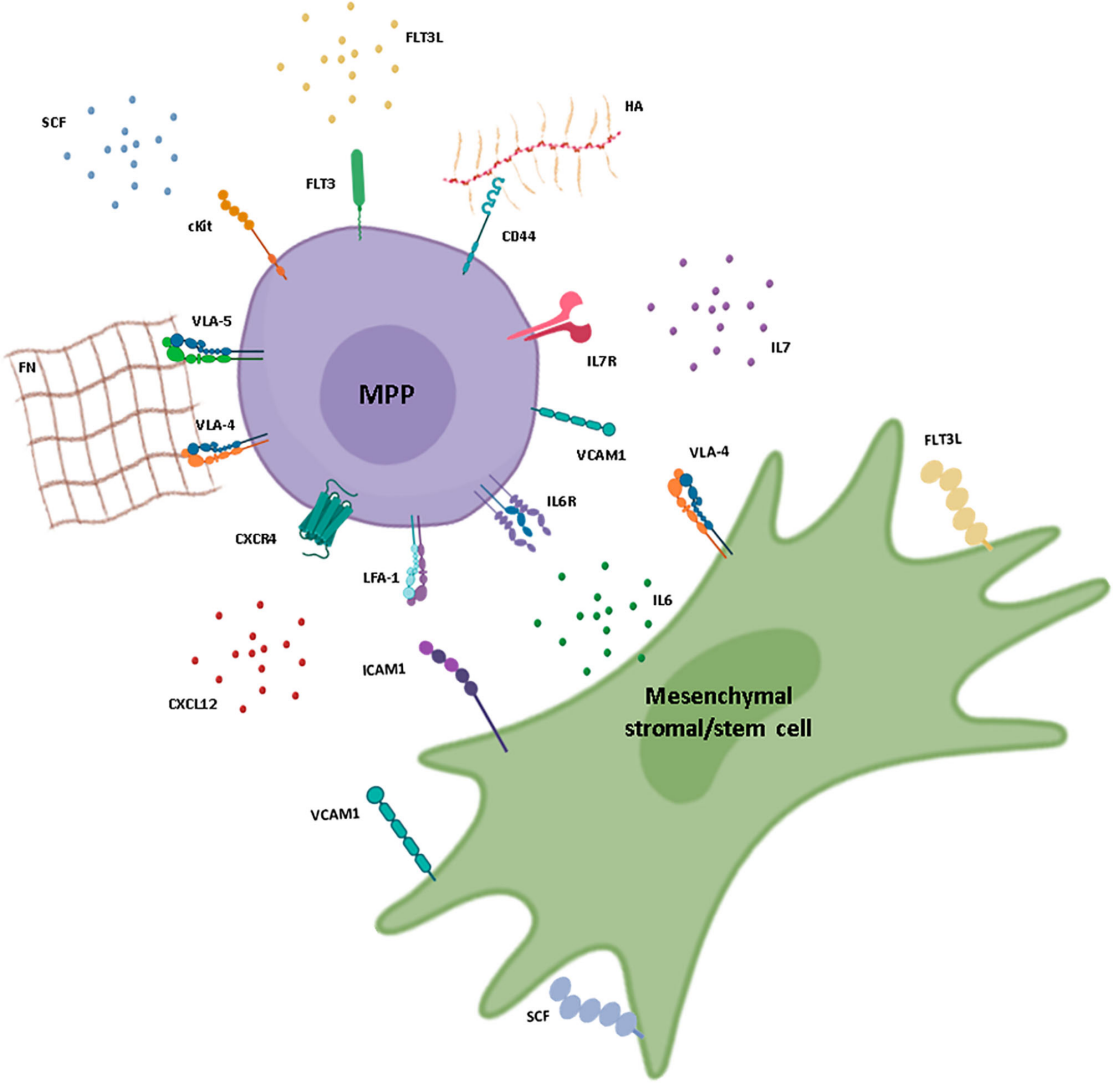
Interactions between hematopoietic multipotent progenitors and mesenchymal stromal/stem cells in the BM. Schematic representation of a multipotent progenitor (MPP) in its BM environment and the possible interactions established with a mesenchymal stromal/stem cell (MSC). Membrane receptors, adhesion molecules, and soluble niche factors previously described for hematopoietic stem and progenitor cell (HSPC) maintenance in the BM are represented. CXCL12, IL-7, IL-6, and SCF are mainly produced by the MSC and bind to CXCR4, IL-7R, IL-6R, and cKit, respectively, at the surface of the MPP. MSCs also produce proteins of the extracellular matrix, such as fibronectin (FN) and hyaluronic acid (HA), which promote cell adhesion through integrin receptors and adhesion molecules expressed by the progenitor cells, such as VLA-4, VLA-5, and CD44. Interactions in the form of cell-cell contacts are also represented and involve VCAM-1 and VLA-4, expressed by both cell types, as well as LFA-1 expressed by MPPs and ICAM-1 expressed by perisinusoidal stromal cells, pericytes, and osteoblasts. Stromal cell factor (SCF) and FLT3L can be found as both soluble and membrane-bound forms. FLT3L is produced mainly by fibroblasts within the BM. IL, interleukin; ILR, interleukin receptor. Figure created using the BioRender icon library.

MPPs are not a homogeneous cell population but are composed of distinct subsets that give rise to more differentiated progenitors in a biased manner in mice. Where these subsets are localized in the BM and how they interact with their environment is still unclear. Distinct lineage-biased precursors seem to express different set of adhesion molecules, with myeloid-biased CD34+ precursors expressing more frequently LFA-1 and L-selectin and expressing a lower surface density of CD44, VLA-4, and VLA-5 than lymphoid-biased precursors (83). Moreover, the Kondo laboratory reported three MPP subsets based on CD135 and Vascular Cell Adhesion Molecule 1 (VCAM-1) expression, which differed in their myeloid potential but displayed similar lymphoid differentiation capacities *in vivo* (84). The difference in adhesion molecule expression pattern may thus correlate with different adhesion capacities and stromal cell niche pairs, which in turn could lead to integration of distinct signals driving cell fate (**Figure 1**). Whether MPP subsets share identical niches is unknown but one can speculate that they establish distinct sets of interactions with their niche even if they share one. A recent study by Balzano et al. showed that HSCs and pro-B cells are frequently found in the same niche in contact with LepR+ PSS cells but form specific interaction networks with these cells, through SCF and CXCL12 for HSCs, and through IL-7 and the basement membrane component Nidogen-1 for pro-B cells (31). If this is also the case for the lineage-biased subsets of MPPs, it could explain the different impact of the CXCL12/CXCR4 axis on myeloid versus lymphoid differentiation (14, 69). Further research is required to address whether MPP subsets reside in distinct niches in the BM and how these niches influence their cell program and fate.

## Plasma Cell Maturation and Maintenance: More Than One Niche?

PCs correspond to the terminal stage of B-cell lineage differentiation and are the effectors of humoral immunity through the secretion of large amounts of Abs. Following antigenic stimulation of their BCRs and/or activation through the Toll-like receptors, B cells can initiate a differentiation process leading to PC generation (85, 86). *In vivo*, this process occurs in two waves. The first wave is called extrafollicular, is very rapid, and leads to PC differentiation in the next few days after antigen exposure (87–89). By contrast, the second wave is delayed, depends on T-cell help, and relies on the formation of germinal centers, which are an anatomical structure within secondary lymphoid organs (90). After their generation in the secondary lymphoid organs, the vast majority of PCs dies rapidly, with a half-life ranging from a few days to a few weeks at most; however, a small pool of PCs persists in the BM for many years, potentially throughout the life of the individual (91–94). Germinal center-derived PCs are “tailored-made” to be the most efficient cell type against the infectious agent, and considered as constituting the main pool of long-lived PCs that persist in the BM.

After their formation in the secondary lymphoid organs, PCs are still immature and are referred to as plasmablasts or short-term PCs. Upon their migration to the BM, they terminate their maturation to become fully differentiated long-term PCs (95). There is still some confusion in the field about the nomenclature that should be used to refer to this intermediate stage of differentiation. The terms plasmablast or short-lived PCs are used interchangeably by some authors, whereas others reserve the term plasmablasts to *in vitro*-differentiated cells, which are very immature compared to PCs obtained *in vivo* after antigen exposure. These potential discrepancies are due to the lack of proper markers for these different stages of PC maturation, especially in mouse models. Of note, the recently published combination of the CD138, TACI, B220, and CD19 markers offers a new scope to discriminate mouse PCs based on their maturation stage (96). In human, the combination of CD19 and CD138 allows to distinguish three steps of PC maturation with the most mature PCs expressing CD138 and losing the expression of CD19 (97). B-cell differentiation into PCs starts in secondary lymphoid organs and is marked by the shunting of B-cell gene expression program. However, the initial event that allows switching from B cells to PCs is not well understood although a decrease in BCR signaling seems an absolute prerequisite for PC differentiation (98).

Newly generated immature PCs upregulate the expression of the transcriptional factor Klf2, which in turn promotes the expression of the S1P1 receptor essential for PC egress from the secondary lymphoid organs and their migration toward the BM through blood circulation (99–104). Immature PCs express the chemokine receptors CXCR4 and CXCR3 at their surface, essential for their migration into the BM and inflammatory sites, respectively (105, 106). Extravasation of PCs from blood to BM through the sinusoids is still poorly described (107) but may represent a key step allowing PC exit from the cell cycle and final maturation, which is characterized by the loss of the B-cell markers B220 and CD19 (96, 97). Once in the BM, the expression of S1P1 and CXCR3 is downregulated in PCs, while the expression of CD138, CD93, and CXCR4 is increased (103, 105, 106, 108–111). CXCR4 is essential for PC homing and, likely retention within the BM as CXCR4-deficient PCs fail to accumulate in this organ (108). Moreover, fine-tuning of CXCR4 signaling is also critical for PC homeostasis in the BM, because a CXCR4-gain-of-function is associated with aberrant accumulation of immature PCs and decreased detection of germinal center-derived antigen-specific PCs in the BM (112, 113).

Once in the BM, PCs finish their differentiation, while possibly losing their ability to migrate (101) and the last remnants of their B-cell identity, including the expression of the B cell co-receptors B220 and CD19 (96, 97, 114–116). Loss of surface expression of BCR was long considered as a hallmark of PC differentiation. However, several reports suggest that some membrane BCR remain expressed at the surface of IgM+, and perhaps IgA+, PCs and continue to induce signaling in PCs (117, 118). PC terminal maturation is also associated with the expression or re-expression of some markers that might reflect their degree of maturity including CD138 in human, and CD28, CD38, and CD93 in mouse (97, 110, 119, 120). Within the BM, PCs stop cycling and become quiescent. Their long-term maintenance depends on factors produced by specific microenvironments, also called niches, whose functions are still not fully understood.

Many studies have demonstrated the key role of BM stromal cells in the maintenance of PCs, partially through their ability to produce CXCL12 (57, 121). Notably, CXCL12+ stromal cells appear essential for PC maintenance, at least *in vitro*. The recent identification of BM stromal cell diversity is just starting to be integrated into the field of PC research; early work just referred to “MSCs” without any further characterization. These “MSCs” likely represent heterogeneous stromal subsets rather than *bona fide* mesenchymal stem cells (101, 122–124). As mentioned before, all stromal cells may not secrete CXCL12, and PSS cells (sometimes referred to as CXCL12-abundant reticular cells) are one of the main sources of this chemokine in the BM (17, 57, 65, 125). Accordingly, BM PCs were reported to be in close contact with perivascular CXCL12+ stromal cells (57, 126, 127), but further characterization of this presumed dialogue is still lacking.

**Figure 2:**
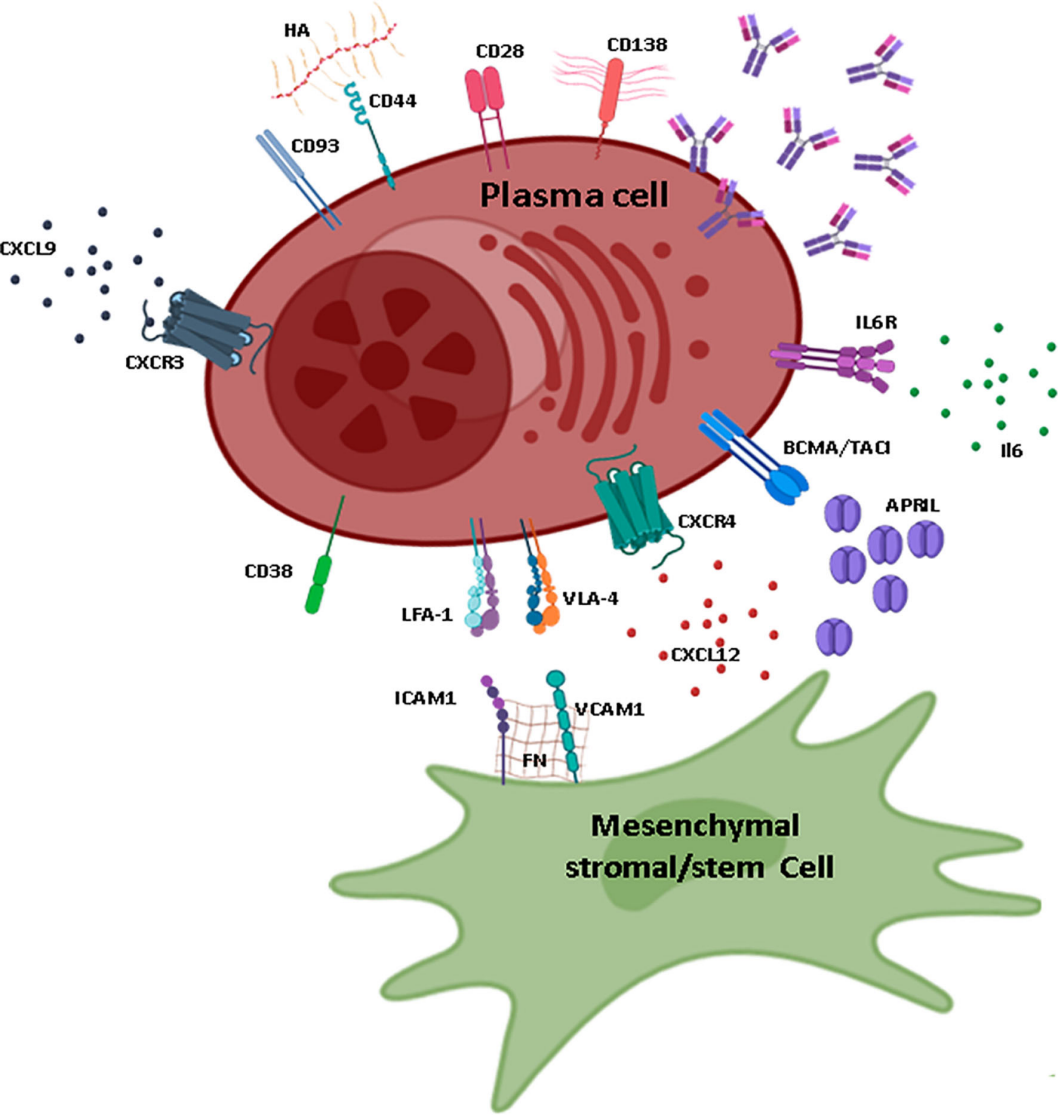
Plasma cell maintenance in the BM. Schematic representation of the main elements necessary for plasma cell (PC) maintenance in the BM, mainly provided by interactions with MSCs. MSCs that sustain PC maintenance are characterized by the production of CXCL12 and are probably CXCL12-abundant reticular cells or PSS subpopulations. MSCs express VCAM-1 and FN that interact with VLA-4 and LFA-1. The main sources of APRIL and IL-6 are cells of myeloid lineage (especially megakaryocytes and eosinophils for APRIL and megakaryocytes, granulocytes, and eosinophils for IL-6). The ligands for CD38 and CD28 are expressed by other cell types and are not represented on this figure. The ligand of CD93 is unknown; however, this adhesion molecule is essential for the maintenance of long-lived PCs. CXCL9, one of the ligands of CXCR3, is produced by osteoblasts. CD138 is one of the main markers for PCs and is characterized by long heparan sulfate chains that trap molecules and allow interactions with the extracellular matrix. HA, hyaluronic acid; FN, fibronectin; IL, interleukin; ILR, interleukin receptor. Figure created using the BioRender icon library.

The close contact established between stromal cells and PCs, notably through integrins and their ligands should be considered as well. Several recent studies have characterized fibronectin as part of the culture-expanded MSC secretome, with an important role in the final maturation and survival of PCs through interaction with the receptor VLA-4 expressed by PCs (102, 122, 124, 128, 129). VLA-4 also interacts with VCAM-1 that is expressed by stromal cells, thus reflecting an important redundancy in adhesion mechanisms (121) (**Figure 2**). Similarly, YWHAZ was found in the human MSC secretome and shown to be essential for PC survival and maturation, potentially through the downregulation of mTORC1 (124). LFA-1 through its interaction with ICAM-1 is also essential, but not sufficient, for PC maintenance because disruption of the LFA-1 signaling axis only causes a transient loss of PCs in the BM (130). *In vitro* experiments have also unraveled a major role for the adhesion molecule CD44, which is highly expressed by BM PCs, for their maintenance. Hyaluronic acid, the ligand of CD44, is a component of the extracellular matrix and the signals induced through CD44 are important to support PC survival (122, 131).

Moreover, PCs require soluble factors for their maintenance. Among them, IL-6 produced notably by eosinophils and stromal cells in BM supports PC survival *in vitro*, although its role *in vivo* is unclear (131–134). PCs also need specific factors for their long-term survival, including TNFα and two cytokines, BAFF and APRIL (135, 136). They express the BR3 (also called BAFF-R), BCMA, and TACI receptors for these cytokines. APRIL is critical, both *in vitro* and *in vivo*, for the maintenance of PCs through the induction of the pro-survival factor Mcl1 (137). In the BM, APRIL is mainly produced by myeloid cells (133, 138–140). Although eosinophils were first reported to be essential for PC maintenance in the BM, it is now accepted that they may play a redundant role in PC maintenance, and that other cell types including megakaryocytes, osteoclasts, monocytes, and even maybe regulatory T cells may contribute to PC survival niches (141, 142). Altogether, these data suggest the existence of a multicellular niche for PCs within the BM with several hematopoietic components and at least one stromal component that may correspond to a CXCL12+ osteoprogenitor. Whether the composition of these survival niches differs between human and mouse is still unknown.

Most of these studies have not discriminated between fully differentiated long-lived PCs and newly produced immature ones. Although the phenotypic changes occurring in PCs as they mature suggest that immature and fully differentiated PCs have different needs for survival factors, further research is needed to understand the actual mechanism. Moreover, the exact location of PCs depending on their maturation stage has never been assessed. The existence of distinct niches or of a distinct set of interactions within a similar niche for PCs according to their maturation stage will require further investigation. In light of a recent paper suggesting that PCs may actually exit the BM and recirculate (143), it would be interesting to determine whether, like for HSPCs, distinct niches control the quiescence and the reactivation of PCs. Finally, BM PC plasticity, motility, and effector functions through Ab and cytokine secretion also need to be considered in the context of the dialogue established with their niche(s) (23). Indeed, through their localization and persistence, PCs may play an important role in the maintenance of HSPC niches.

## Concluding Remarks and Unanswered Questions

**Figure 3:**
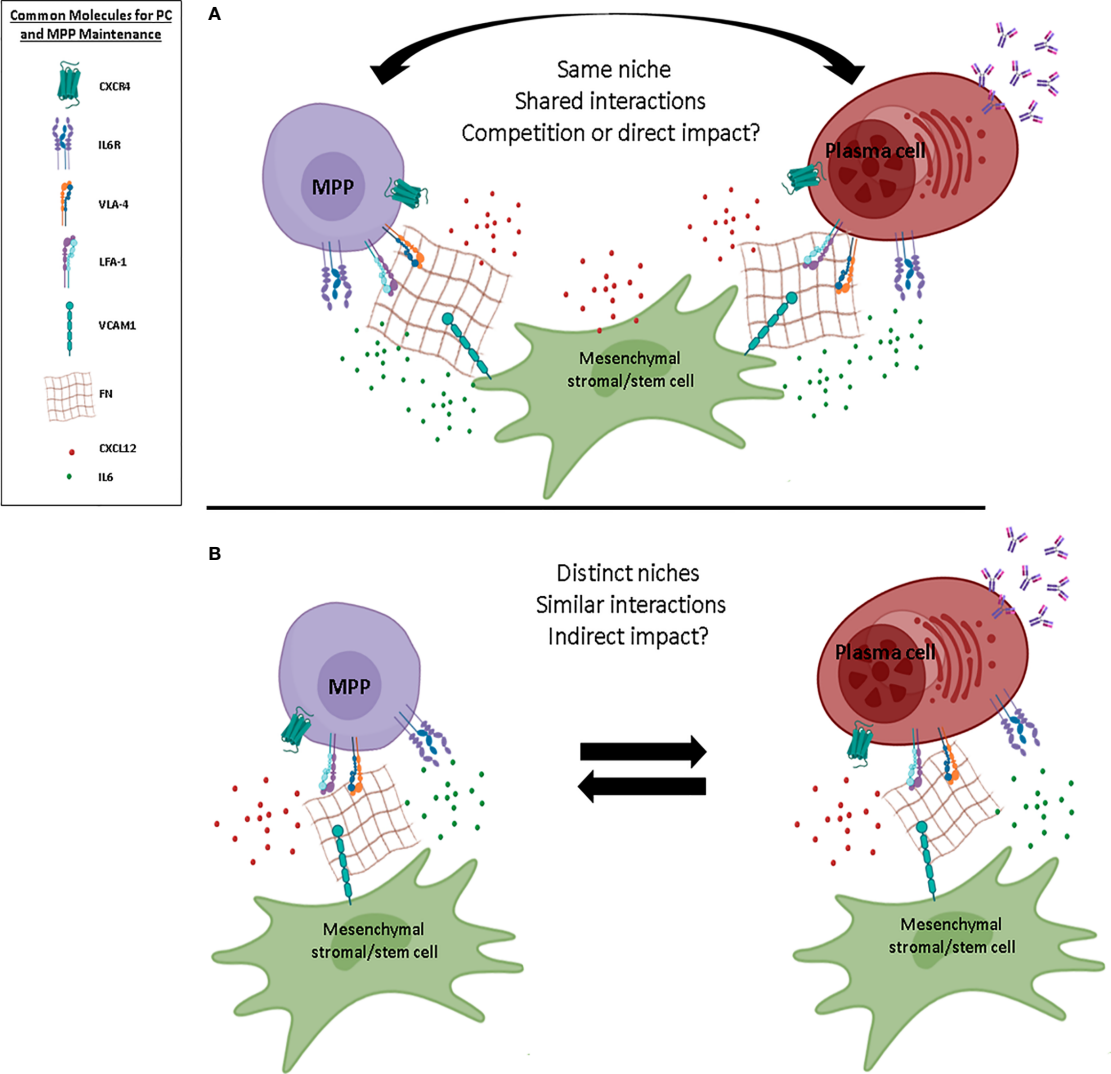
Possible BM niche models for MPPs and PCs. Schematic representation of two different models of multipotent progenitor (MPP) and plasma cell (PC) niches within the BM. **(A)** In the first model, MPPs and PCs share common niches and reciprocally impact their biology, maintenance, and fate. **(B)** In the second model, MPPs and PCs display distinct niches despite common ligand/receptor interactions, and potentially have an indirect impact on each other. Common molecules for PC and MPP maintenance within the BM are shown. FN, fibronectin; IL, interleukin; ILR, interleukin receptor. Figure created using the BioRender icon library.

Although our understanding of the elements necessary for the maintenance of MPPs and PCs within the BM has tremendously improved during recent years, several open questions remain. For instance, the localization of the different MPP subsets remains unknown. Although PCs are found mainly in close contact with CXCL12+ cells, the nature of these cells and how they drive the precise positioning of PCs in the BM is not well understood. Whether PC localization changes during their final maturation is also unclear. As highlighted in this review, several factors of the BM niches are essential for the maintenance of MPPs and PCs. Both cell types share common characteristics including adhesion molecules such as CD44 and LFA-1, and dependence on cytokine/chemokine such as CXCL12 and IL-6. These similarities, together with the ability of MSCs to produce some of the common factors and to support them *in vitro*, may argue in favor of a unique niche able to maintain both MPPs and PCs (**Figure 3A**). If true, whether MPPs and PCs share or compete for these niches is an open question. Furthermore, it is worth noting that in conditions where the BM homeostasis is disrupted (*e.g.*, inflammation, aging), PCs can indeed impact HSPCs and MSCs. It is known that both myelopoiesis and the number of PCs increase with aging in the BM. Recently, two studies reported that PC accumulation with age regulates the production of inflammatory factors by BM stromal cells, which in turn promotes myeloid-biased HSCs (144, 145). This impact on myeloid cells is probably due to the ability of PCs to produce IL-10, one of the key drivers of the myeloid differentiation (145, 146). Consequently, both studies suggest a potential impact of PCs on MSCs and on the skewing of MPPs toward myeloid lineage with age (10). Moreover, external factors, such as dietary restriction and exercise, instruct hematopoietic precursors and mature lymphocytes *via* modulation of BM stromal cells (147, 148). Interestingly, in Multiple Myeloma, that is characterized by a massive influx of malignant PCs in the BM, the architecture of the BM is disorganized with numerous lytic bone lesions and the hematopoiesis process is also frequently impaired. Myeloma PCs alter the function of osteoclasts and adipocytes to support their maintenance, through their ability to produce fatty acid and growth factors like IL6 or TNF-α (149–153). In this context, malignant PC-imprinted BM stromal cells support deregulation of the HSPC compartment, suggesting that hematopoietic dysfunction in Multiple Myeloma results from PC-related microenvironmental alterations (154, 155). While the impact of PCs on HSPCs seems clear in pathological settings, it is still unknown whether PCs and MPPs may affect each other in the “young” BM and at steady state.

Based on our current understanding of the BM ecosystem, we cannot affirm that the maintenance of PCs and MPPs is carried out by only one cell subpopulation of stromal cells. If the PSS cells are a good candidate due to their strong expression of CXCL12 and their perivascular localization, this cell population is outnumbered by MPPs and PCs, which, albeit rare, are still about 10 times more numerous than PSS cells. This simple observation questions a model wherein cells may form exclusive pairs in their niche. Furthermore, PCs and MPPs are both heterogeneous populations. At least two populations of PCs (based on their maturation status) and three distinct subpopulations of MPPs have been described in the BM. It is difficult to envision how these different subsets may coexist in common niches. Moreover, long-lived PCs are terminally differentiated and relatively quiescent cells, in contrast to MPPs, which can cycle and differentiate, suggesting that both the MPP and PC pools display a differential turn-over. Such restrictions suggest another model with two distinct niches for MPPs and PCs, featuring common factors and with some possible exchange and/or interactions. However, such niches have not been characterized so far and more insights into the relative functions of the heterogeneous MSC subsets will be necessary to support this model (**Figure 3B**). In conclusion, the two models proposed are equally possible and are not mutually exclusive; indeed, the existence of dynamic interactions between the BM, inter-, and/or intra-niches is probably the closest to reality. This may be linked to the ability of the cells to come and go and, consequently, to modulate their niche to fit their own needs.

## Author Contributions

AB and JPL made extensive review of the literature listed, drafted different sections of the review, and drawn the figures. ME and KB conceived, designed, and supervised the review. All authors contributed to the article and approved the submitted version.

## Funding

This study was funded by the ANR (grants ANR-19-CE15-0019-01 and ANR-17-CE14-0019), a “Fondation ARC pour la recherche sur le cancer” grant, an INCa grant (PRT-K 2017), the Association Saint Louis pour la Recherche sur les Leucémies, a grant from IdEx Université de Paris (ANR-18-IDEX-0001) and the People Program (Marie Curie Actions) of the European Union’s Seventh Framework Program (FP7/2007-2013) under REA grant agreement. PCOFUND-GA-2013-609102 through the PRESTIGE program coordinated by Campus France.

## Conflict of Interest 

The authors declare that the research was conducted in the absence of any commercial or financial relationships that could be construed as a potential conflict of interest.

## Abbreviations

Ab, antibody; BCR, B Cell Receptor; BM, bone marrow; CLP, common lymphoid progenitor; EC, endothelial cells; Emcn, Endomucin; GPCR, G protein-coupled receptor; HSC, hematopoietic stem cell; HSPC, hematopoietic stem and progenitor cells; LSK, Lin-Sca1+c-kit+; LT-HSC, long-term hematopoietic stem cell; MPP, multipotent progenitor; MSC, mesenchymal stromal/stem cell; PC, plasma cell; PSS, perisinusoidal stromal cell; PVS, perivascular cell; SCF, stem cell factor; ST-HSC, short-term hematopoietic stem cell; vWF, von Willebrand factor; WHIM, warts, hypogammaglobulinemia, infections, and myelokathexis; VCAM1, Vascular Cell Adhesion Molecule 1.
